# Factors associated with dental caries among institutionalized residents with schizophrenia in Taiwan: a cross-sectional study

**DOI:** 10.1186/1471-2458-10-482

**Published:** 2010-08-13

**Authors:** Kuan-Yu Chu, Nan-Ping Yang, Pesus Chou, Hsien-Jane Chiu, Lin-Yang Chi

**Affiliations:** 1 Community Medicine Research Center & Institute of Public Health, National Yang-Ming University, Taipei, Taiwan; 2 Department of Dentistry, Taipei County Hospital, Taipei County, Taiwan; 3 Department of Geriatrics, Tao-Yuan General Hospital, Department of Health, Taoyuan, Taiwan; 4 Department of Psychiatry, Jia-Nan Mental Hospital, Department of Health, Tainan, Taiwan; 5 Department of Dentistry, National Yang-Ming University, Taipei, Taiwan; 6 Department of Dentistry, Taipei City Hospital, Taipei, Taiwan

## Abstract

**Background:**

Little research has been done on the relationship between dental caries and the personal characteristics of institutionalized residents diagnosed with schizophrenia. This study investigates the individual and treatment factors associated with the dental caries among institutionalized residents with schizophrenia in Taiwan.

**Methods:**

An oral health survey of institutionalized residents with schizophrenia in the largest public psychiatric hospital was conducted in Taiwan in 2006. Based on this data, multiple logistic analyses were used to determine the relationship between some explanatory variables and the outcome variables of dental caries among subjects with schizophrenia.

**Results:**

Among the 1,108 subjects with schizophrenia, age was the only variable independently associated with DMFT > 8 (OR = 7.74, 95% CI = 3.86-15.55, p < 0.001 in comparison to residents aged 65 + years vs. 20-44 years; OR = 3.06, 95% CI = 2.03-4.61, p < 0.001 in comparison to residents aged 55-64 years vs. 20-44 years) after making adjustments for other explanatory variables. In addition, those with an education of only elementary school (OR = 1.67, 95% CI = 1.08-2.56, p = 0.021), low income (OR = 1.58, 95% CI = 1.02-2.44, p = 0.039), and length of stay (LOS) of > 10 years (OR = 2.09, 95% CI = 1.30-3.37, p = 0.002) were associated with a care index < 54.7%. Older age, lower educational level, and longer hospital stays were associated with number of remaining teeth being < 24.

**Conclusions:**

Aging was the most important factor related to a high level of dental caries. Low educational level, low income, and LOS were also associated with the indicators of dental caries among institutionalized subjects with schizophrenia. It is necessary to address the treatment factors such as prolonged stay in institutions when decision-makers are planning for preventive strategies of oral health for institutionalized residents with schizophrenia.

## Background

Factors such as age and the length of stay (LOS) in institutions seem to be associated with the dental caries of psychiatric patients [1-4]. Schizophrenia is the most complex psychiatric disorder that affects mankind; treatment of institutionalized residents, especially those with schizophrenia, takes up a significant part of the health care resource, compared to other psychiatric inpatient groups [5,6]. However, to date, there has been relatively little research conducted on factors associated with dental caries among this population.

In addition to age and LOS, there were many other possible factors associated with dental caries in patients with schizophrenia. An early study showed that women with schizophrenia were at a higher risk than men in terms of dental caries [1]. In addition, the level of dental caries among patients was related to the severity of their schizophrenia [7,8]. A treatment factor, the first generation anti-psychotics (FGA), which are part of a wide array of medications used for schizophrenia treatment, may cause profound hypo-salivation by blocking the parasympathetic stimulation of the salivary glands; this intensifies the progression of caries [8-11].

To focus on the patients with schizophrenic disorder and adjust for explanatory confounders, this study included a large number of subjects, aiming to investigate the individual and treatment factors associated with dental caries among institutionalized residents with schizophrenia in Taiwan.

## Methods

### Subjects

A cross-sectional oral health survey was conducted in Yuli Hospital, Department of Health (DoH), Taiwan. This hospital admits the largest number of patients among all public psychiatric institutions in Taiwan. The study subjects included institutionalized residents with the main psychiatric diagnosis of schizophrenic disorders, as categorized by the International Classification of Disease (9^th ^Edition) (ICD-9 code 295). The participants completed an oral health examination in accordance with the protocol of World Health Organization (WHO) [12]. Examinations were performed under standard conditions. A trained dentist and an experienced assistant completed the dental caries examinations. Ethical clearance was issued by the Ethical Committee of Yuli Hospital, DoH, Taiwan. As of July 2006, the hospital had admitted 1,468 patients with schizophrenia. A total of 1,108 subjects (response rate of 75.5%) completed the dental caries examinations. In this survey, decayed, missing and filled teeth index (DMFT) for each subject was calculated to describe the experience of dental caries. The care index (CI) was defined as the ratio of the number of filled teeth to the number of DMFT. The number of remaining teeth (NRT) of the subjects was also individually assessed.

The mean age of the 1,108 institutionalized subjects with schizophrenia was 50.8 years (standard deviation/SD = 10.8). Of these, 73% were men. The patients had stayed in this hospital for 8.4 years on average (SD = 5.7). The mean score of DMFT was 13.9 (SD = 8.5), CI 14.3% (SD = 26.5), and NRT 17.7 (SD = 8.8).

### Outcome variables

The dependent variables of this investigation were the categorical outcome variables of dental caries. They were based on the hypothesis that institutionalized residents with schizophrenia should have a comparable level of oral health status to the general population. We chose the cut-off points of the dental caries indexes as the corresponding means from an oral health survey for general Taiwanese [13]. The survey involved 2,660 adults who completed oral health examinations using methods suggested by WHO. Results showed that the mean number of DMFT was 8.4; the CI was 54.7%; and the average number of NRT was 23.2. The categorical outcome variables of dental caries were then set to: DMFT > 8, CI < 54.7%, and NRT < 24.

### Explanatory variables

The explanatory variables, which represent personal characteristics, were individual factors (such as sex, age, educational level, marital status, grade of disability, and economic status), and treatment factors (such as use of anti-psychotics and LOS). Using chart reviews, the personal characteristics of the subjects were recorded.

Age was classified in 4 strata: 20-44, 45-54, 55-64, and 65 years old and over (65+). Educational level was stratified into 3 categories: high school level and higher (high school+), elementary school, and no schooling. Marital status was stratified into 3 categories: single, married, and separated/divorced/widowed. The grade of disability, which was recorded using the disability certificate issued by the Ministry of the Interior in Taiwan, included mild/moderate (moderate), severe, and profound. Economic status was categorized as low income or non-low income, depending on the low-income certificate issued by local authorities. Anti-psychotics used during the last 6 months prior to the survey were comprised of FGA (typical or conventional antipsychotics, including Rosup, Haloperidol, Halin, Fluanxol, Clopixol, Mellazine, Sulpyride F.C., Surin, U-Dolan, Morefine, and Betamac), and non-FGA. Non-FGA included the second generation anti-psychotic (SGA or atypical anti-psychotics, such as Mezapin, Uspen, Clopine, Zyprexa, Lodopin, Risperdal, and Seroquel), and the third generation anti-psychotic (TGA, such as Abilify). Meanwhile, the cut-off point for the LOS, which refers to the number of years since admission to the Hospital, was 10 years [8].

### Analysis

Chi-square test was used to examine the explanatory factors for the outcome variables, specifically, DMFT > 8, CI < 54.7%, and NRT < 24. Three multivariate logistic models were developed to identify the most informative factors related to the outcome variables. The explanatory variables with a cut-off of p-value < 0.1 from univariate analyses were used and included into the multi-variable logistic models. The odds ratio (OR) of the explanatory variables compared to the reference category, the significance (p-value), and the 95% confidence intervals (95% CI) were estimated from multiple logistic regression models. Areas below the receiver-operating characteristics (ROC) curve were also calculated for the measurement of the predicted probability of the logistic models. Database management and statistical analyses were performed using the SPSS software.

## Results

### Univariate analysis results

The distribution of subjects and results of the univariate analyses are shown in Table 1. The DMFT > 8 was significantly related to independent variables such as age, educational level, grade of disability, and LOS. The CI < 54.7% was significantly related to sex, age, educational level, grade of disability, economic status, and LOS. The NRT < 24 was significantly related to age, educational level, grade of disability, use of anti-psychotics, economic status, and LOS.

**Table 1:** Distribution and univariate analysis between explanatory variables and categorical outcome variables of dental caries among institutionalized subjects with schizophrenia.

Variables	Total	DMFT > 8		CI < 54.7%		NRT < 24	
	No. (%)	No. (%)	p-value	No. (%)	p-value	No. (%)	p-value
**Individual factors**							
Sex							
Men	809 (73.0)	542(67.0)	0.127	713(89.8)	0.054	522 (64.5)	0.944
Women	299 (27.0)	215(71.9)		254(85.5)		192 (64.2)	
Age							
20-44	302(27.3)	169(56.0)	< 0.001	230(77.2)	< 0.001	122 (40.4)	< 0.001
45-54	426(38.4)	271(63.6)		381(90.9)		272 (63.8)	
55-64	258(23.3)	206(79.8)		235(93.3)		205 (79.5)	
65+	122(11.0)	111(91.0)		121(99.2)		115 (94.3)	
Educational level							
High school+	470(42.4)	296(63.0)	0.002	385(83.0)	< 0.001	257 (54.7)	< 0.001
Elementary school	518(46.8)	369(71.2)		467(91.9)		359 (69.3)	
No schooling	120(10.8)	92(76.7)		115(96.6)		98 (81.7)	
Marital status							
Single	852(76.9)	578(67.8)	0.473	748(89.2)	0.475	542 (63.6)	0.419
Married	116(10.5)	85(73.3)		100(88.5)		81 (69.8)	
Separated etc.	140(12.6)	94(67.1)		119(85.6)		91 (65.0)	
Grade of disability							
Moderate	237(21.4)	147(62.0)	0.035	195(83.0)	0.001	130 (54.9)	< 0.001
Severe	747(67.4)	518(69.3)		655(89.2)		489 (65.5)	
Profound	124(11.2)	92(74.2)		117(95.9)		95 (76.6)	
Economic status							
Non-low income	240(21.7)	164(68.3)	1.000	190(80.9)	< 0.001	138 (57.5)	0.012
Low income	868(78.3)	593(68.3)		777(90.8)		576 (66.4)	
**Treatment factors**							
Use of anti-psychotics							
Non-FGA	694(62.6)	474(68.3)	1.000	602(88.0)	0.431	430 (62.0)	0.027
FGA	414(37.4)	283(68.4)		365(89.7)		284 (68.6)	
LOS							
≤ 10 years	520(46.9)	333(64.0)	0.004	416(81.7)	< 0.001	278 (53.5)	< 0.001
> 10 years	588(53.1)	424(72.1)		551(94.7)		436 (74.1)	

Abbreviations: DMFT = Decayed, Missing and Filled Teeth; CI = Care Index; NRT = Number of Remaining Teeth; FGA = First Generation Anti-psychotics; LOS = Length of Stay

### Multivariate analysis results

Results of multiple logistic regressions for the outcome variables are shown in Table 2. The association between DMFT > 8 and age was the only relationship that remained statistically significant (all p-values being < 0.05). The 3 levels of age were significantly associated with DMFT > 8, with ORs ranging from 1.38 (for 45-54 years) to 7.74 (for 65 + years) compared with the 20-44 years as the reference category. Age was independently associated with DMFT > 8 after an adjustment for other explanatory variables. The area under the ROC curve (AUC) of the multiple logistic regression equation for DMFT > 8 was 0.66.

**Table 2:** Multivariate analyses between explanatory variables and categorical outcome variables of dental caries among institutionalized subjects with schizophrenia.

Variables	DMFT > 8^a^		CI < 54.7%^b^		NRT < 24^c^	
	OR (95% confidence interval)	p-value	OR (95% confidence interval)	p-value	OR (95% confidence interval)	p-value
**Individual factors**						
Age						
20-44	1 (Reference)		1 (Reference)		1 (Reference)	
45-54	1.38 (1.01-1.90)	0.044	2.19 (1.40-3.44)	0.001	2.26 (1.65-3.10)	< 0.001
55-64	3.06 (2.03-4.61)	< 0.001	2.19 (1.19-4.04)	0.012	4.27 (2.84-6.43)	< 0.001
65+	7.74 (3.86-15.55)	< 0.001	14.37 (1.91-108.05)	0.010	16.29 (7.15-37.10)	< 0.001
Educational Level						
High school+			1 (Reference)		1 (Reference)	
Elementary school			1.67 (1.08-2.56)	0.021	1.34 (1.01-1.79)	0.045
No schooling			2.62 (0.90-7.61)	0.076	1.67 (0.98-2.86)	0.062
Economic Status						
Non-low income			1 (Reference)			
Low Income			1.58 (1.02-2.44)	0.039		
**Treatment factors**						
LOS						
≤ 10 years			1 (Reference)		1 (Reference)	
> 10 years			2.09 (1.30-3.37)	0.002	1.35 (1.00-1.81)	0.047
**AUC**	0.655		0.762		0.730	

Abbreviations: DMFT = Decayed, Missing and Filled Teeth; CI = Care Index; NRT = Number of Remaining Teeth; OR = Odds Ratio; LOS = Length of Stay; AUC = Area Under the ROC (Receiver-Operating Characteristics) Curve

a. Variables entered: Age, Educational level, Grade of disability, and LOS

b. Variables entered: Sex, Age, Educational level, Grade of disability, Economic status, and LOS

c. Variables entered: Age, Educational level, Grade of disability, Economic status, Use of anti-psychotics, and LOS

In addition, age, education (elementary school), economic status, and LOS were all significantly associated with CI < 54.7%. From the results, those in the older age groups, with elementary schooling (adjusted OR = 1.67, 95% CI = 1.08-2.56, p = 0.021), low income (adjusted OR = 1.58, 95% CI = 1.02-2.44, p = 0.039), and LOS over 10 years (adjusted OR = 2.09, 95% CI = 1.30-3.37, p = 0.002) were more likely to have CI < 54.7% compared to the respective reference category. The AUC for CI < 54.7% was 0.76.

Educational level and LOS were also significantly related with NRT < 24. Those in the older age groups, with elementary school education (adjusted OR = 1.34, 95% CI = 1.01-1.79, p = 0.045), and LOS > 10 years (adjusted OR = 1.35, 95% CI = 1.00-1.81, p = 0.047), were more likely to have NRT < 24 compared with the respective reference category. The AUC for NRT < 24 was 0.73.

## Discussion

Data from a large sample of institutionalized residents with schizophrenia were used to explore the relationships between personal characteristics and dental caries indexes of the subjects. Results of this study showed that DMFT was associated with the age of subjects and none of the other variables explored. Lower educational level and longer stay in hospital were related to CI and NRT. Low economic status was another important factor related to CI. However, we did not find that the use of anti-psychotics was associated with dental caries indexes. These findings may provide new thinking for health policy decision-makers and oral health professionals to plan more effective oral health improvement strategies for this specific population [14].

Age was the only explanatory variable entered into the final logistic model for DMFT > 8 in this study. In fact, age is a strong confounder of the DMFT score, overcoming the effects of other explanatory variables. This is reasonable because co-linearity was significant between educational level, grade of disability, LOS, and age (all p-values < 0.001 for the Pearson correlation test) in this study. This result was consistent with findings in Italy, India, and Israel [7,15,16].

Our results also showed that the factors associated with CI of the schizophrenic subjects were older age, lower educational level, low income, and longer stay in institutions. Schizophrenia seems to be more common in poor families, but their economic status lessens the chance of receiving adequate dental care, which could partly explain the results [6,17,18]. Our results, which indicated that age was correlated with the NRT, are similar to those found in Israel [19], although we also found that the LOS was another important factor for NRT.

Our results indicated that a longer LOS was associated with increased risks of CI and NRT. Since the psychiatric health care system has not yet been fully established in Taiwan, patients with schizophrenic still cannot obtain the necessary care in their communities. Individuals suffering from severe schizophrenia may be able to attain a more dignified life if they could avail themselves of personalized, private, and high-quality care services in pertinent institutions. To stay in long-term care institutions is, perhaps, the alternative solution to living in the community. Therefore, the reform of institutions, particularly for the provision of relevant services and continued care, can compensate for a little imparity of dental care for these patients, and is a more practical solution than de-institutionalization of schizophrenic inpatient care [20-22].

It was difficult to compare participants with non-participants in terms of the personal characteristics influencing the dental caries indexes in this study. In fact, the majority of those who did not participate in the survey were living in a rehabilitation hospice for psychotics with low physical functions or severe negative symptoms of schizophrenia. This symptom was potentially devastating to oral health because it impaired the patient's desire and ability to adhere to preventive oral hygiene [8,23,24]. As a result, this survey probably underestimated the level of dental caries of the study population.

Some limitations of this study should be noted. First, the enrolled subjects were not randomly sampled from all institutionalized residents with schizophrenia in Taiwan. Thus, generalization of the results to populations with different backgrounds may be limited. Second, some other possible confounders, e.g. general health condition, smoking habits, and body weight of the subjects, were not considered due to insufficient information [25-27].

## Conclusions

One of the individual factors examined, i.e. older age, had an independent effect on the risk of high DMFT score after adjusting for other explanatory factors. Furthermore, a prolonged stay in institution was the only treatment factor that was found to be related to CI and NRT. These results could provide an improved insight for psychologists and dentists who provide dental preventive services for this element of the population with special needs.

## Competing interests

The authors declare that they have no competing interests.

## Authors' contributions

NPY, HJC and LYC constructed the study protocol under the supervision of PC. KYC performed the oral examinations and data collection. NPY, HJC and LYC performed the statistical analyses and interpretation of the study. KYC drafted the initial manuscript and all authors revised, read and approved the final manuscript.

## Pre-publication history

The pre-publication history for this paper can be accessed here:

http://www.biomedcentral.com/1471-2458/10/482/prepub
